# 我如何治疗复发/转化滤泡性淋巴瘤

**DOI:** 10.3760/cma.j.issn.0253-2727.2023.12.003

**Published:** 2023-12

**Authors:** 兵 徐, 志娟 林

**Affiliations:** 厦门大学附属第一医院血液科，厦门 361003 Department of Hematology, the First Affiliated Hospital of Xiamen University and Institute of Hematology, School of Medicine, Xiamen University, Key Laboratory of Xiamen for Diagnosis and Treatment of Hematological Malignancy, Xiamen 361003, China

滤泡性淋巴瘤（Follicular lymphoma, FL）是一类起源于滤泡中心B细胞的非霍奇金淋巴瘤（NHL），是最常见的惰性淋巴瘤类型。我国FL的发病率占B细胞NHL的8％～23％，低于欧美地区。该病总体预后良好，中国滤泡性淋巴瘤工作组总结中国大样本多中心结果显示5年无进展生存（PFS）率和总生存（OS）率分别为61％和89％[1]。目前FL的主要诊治挑战包括：①约20％患者一线接受免疫化疗后2年内出现疾病进展（POD24），这类患者生存率显著下降，如何早期识别及治疗以改善预后。②进展期FL（Ⅲ/Ⅳ期）具有不可治愈的特点，多线复发患者治疗困难。③8％～70％的FL患者在诊断后15年内会转化为侵袭性淋巴瘤，其中以弥漫大B细胞淋巴瘤（DLBCL）最为常见，年转化发生率为2％～3％，转化FL（tFL）患者预后显著下降。本文拟通过介绍3个典型病例的诊疗经过，分享本中心在FL这些挑战中的诊治经验。

一、早期进展FL的治疗

1. 典型病例：女，46岁。因“颈部、双侧腋窝多发肿物3个月”于2018年12月入院。完善淋巴结活检提示（右腋下）FL，2级。免疫组化：CD20（+），CD10（+），BCL6（+），CD21（滤泡网+），Ki-67（40％+）。骨髓活检提示淋巴瘤累及。PET-CT：淋巴瘤累及全身广泛淋巴结，累及脾脏、全身骨髓可能。诊断FL（2级Ⅳ期A，FLIPI 1分，FLIPI-2 1分），因未达到治疗指征，给予观察等待。2021年6月出现左侧扁桃体肿大，影响吞咽，扁桃体活检病理回报FL，3A级。免疫组化：CD20（+），CD10（+），BCL6（+），CD21（滤泡网+），Ki-67（70％+），BCL2（+）。PET-CT：淋巴瘤累及全身广泛淋巴结，全身骨髓病灶，病灶较前期增多、增大，代谢较前明显增高，最大标准摄取值（SUVmax）达16.7，考虑病情进展。因患者出现压迫症状，达到治疗指征，2021年7月至11月予R-CHOP方案治疗6个疗程，序贯利妥昔单抗（BR）维持。2022年5月患者在维持过程中再发左侧腹股沟淋巴结肿大，病理活检提示FL，3A级。免疫组化：CD20（+），CD21（滤泡网+），BCL2（+），BCL6（+），CD10（+），MUM-1（部分+），Ki-67（50％+）。诊断FL（3A级Ⅳ期A，FLIPI 1分，FLIPI-2 1分），考虑POD24，入组CD20×CD3双特异性抗体Mosunetozumab联合来那度胺（Mos-R）临床研究，目前已给予9个疗程Mos-R方案治疗，3个疗程后评估达到完全缓解（CR）并持续至今，取得满意疗效。

2. 早期进展FL的早期识别：根据中国真实世界研究，POD24人群约占全部FL人群的20.7％，这组人群的5年OS率相比非POD24人群显著下降（72％对96％，*P*<0.001）[1]。如何早期识别POD24是目前比较大的挑战。目前有多种预后模型来预测FL患者的预后（表1）。其中临床模型包括FLIPI、FLIPI-2、PRIMA-PI以及FLEX[2]–[5]。FLIPI、FLIPI-2及PRIMA-PI相对简单易行，但这些模型主要是预测PFS和OS，对POD24预测的敏感性和特异性不能满足临床需求，FLEX模型对预测POD24的敏感性略高于其他3个模型，但临床可操作性相对较差。值得注意的是，上述临床模型均是基于免疫化疗的临床研究，尚不适用于CD20单抗联合来那度胺的无化疗方案。而分子遗传学因素与临床因素进行整合的预后模型为m7-FLIPI、POD24-PI及23基因模型等[6]–[8]。然而，以上模型具有以下不足：①不同基因模型涉及的基因差别巨大，可能导致不同模型预测的危险度不同；②因为经济等问题，大部分患者未行分子遗传学检测，导致分子模型不易推广；③上述模型同样是基于免疫化疗的临床研究，尚不适用于CD20单抗联合来那度胺的方案。为更加准确预测POD24，2022年Maurer等[9]提出新的预后模型FLIPI24，包括5个连续变量（年龄、血红蛋白、白细胞计数、乳酸脱氢酶和β_2_-微球蛋白），可较好地预测不同风险组患者的24个月无事件生存（EFS24）以及OS。在内外部验证中，FLIPI24预测EFS24和OS的性能均优于目前的临床预后模型。总体而言，目前尚无理想预后模型可精准预测POD24。从数据上分析FLIPI24相对较好，值得进一步临床验证。

**表1:** 滤泡性淋巴瘤的预后模型

预后模型	项目	分层	预后影响	高危组预测POD24的敏感性	高危组预测POD24的特异性
FLIPI[2]	年龄>60岁；Ⅲ～Ⅳ期；	低危（0～1分）	5年OS率	53%～78%	56%～62%
	血红蛋白<120 g/L；	中危（2分）	91%（低危）		
	LDH>正常值；	高危（≥3分）	78%（中危）		
	>4个淋巴结区受累		53%（高危）		
FLIPI-2[3]	年龄>60岁；骨髓累及；	低危（0～1分）	5年OS率	53%	59%～76%
	血红蛋白<120 g/L；	中危（2分）	80%（低危）		
	β_2_微球蛋白>正常值；	高危（≥3分）	51%（中危）		
	单个受累淋巴结>6 cm		19%（高危）		
PRIMA-PI[4]	β_2_微球蛋白>3 g/L；	低危（0分）	5年FFS率	69%	48%
	骨髓累及	中危（1分）	69%（低危）		
		高危（2分）	55%（中危）		
			37%（高危）		
FLEX[5]	男性；FL 3a；	低危（0～2分）	3年PFS率	60%	68%
	病灶直径总和为最高4分位；	高危（3～9分）	86%（低危）		
	>2个结外部位受累；		68%（高危）		
	ECOG评分>2；				
	血红蛋白<120 g/L；				
	β_2_微球蛋白>正常值；				
	LDH>正常值；				
	NK细胞>100 /µl				
M7-FLIPI[6]	ECOG>1；	低危	5年FFS率	43%～61%	77%～86%
	FLIPI评分高危；	高危	68%~77%（低危）		
	突变（EP300，CREBBP, CARD11，MEF2B, EZH2，ARID1A, FOXO1）		22%~38%（高危）		
POD24-PI[7]	ECOG>1；	低危	5年FFS率	54%～78%	67%～73%
	FLIPI评分高危；	高危	72%~77%（低危）		
	突变（EP300，EZH2，FOXO1）		36%~50%（高危）		
23基因模型[8]		低危	5年PFS率	43%	79%
		高危	56%（低危）		
			26%（高危）		

注 POD24：2年内疾病进展；OS：总生存；PFS：无进展生存；FFS：无治疗失败生存；LDH：乳酸脱氢酶；ECOG：美国东部肿瘤协作组体能评分；FLIPI：滤泡淋巴瘤国际预后评分；FL：滤泡性淋巴瘤

3. 早期进展FL患者的治疗选择：目前POD24尚无统一规范的治疗。一项回顾性分析探讨POD24人群接受自体造血干细胞移植（auto-HSCT）和未接受auto-HSCT的生存差异。研究显示，auto-HSCT有改善POD24患者生存的趋势，移植组5年OS率为67％，而非移植组为60％。亚组分析还显示，在治疗结束1年内进展的患者，auto-HSCT可使患者生存率显著提高至73％[10]。而另一项德国的回顾性分析则显示，auto-HSCT可显著提高患者5年OS率（77％对59％，*P*＝0.031）[11]，因此，POD24患者再次获得缓解后推荐行auto-HSCT巩固治疗。

对于不适合移植或化疗不敏感人群，可考虑靶向药物，如来那度胺、磷脂酰肌醇3-激酶（PI3K）抑制剂及EZH2抑制剂等。MAGNIFY研究探讨BR联合来那度胺（R2）方案序贯维持治疗在难治复发FL（R/R FL）患者中的疗效。研究显示，在POD24患者中，R2方案总反应率（ORR）为48％，CR率仅为12％；相比总人群的ORR 67％和CR率36％仍有下降[12]。目前中国推荐用于R/R FL的2种PI3K抑制剂包括度维利塞及林普利塞。亚组分析提示PI3K抑制剂治疗POD24人群的ORR约为33％，中位疗效持续时间（DOR）为12.6个月，与非POD24人群类似[13]。同时，PI3K抑制剂的安全性值得关注，本中心报道PI3K抑制剂不良反应的meta分析[14]，入组4项临床研究包含1 399例患者，显示PI3K抑制剂显著增加严重不良反应的发生率（*P*<0.05）。其中粒细胞缺乏、肺炎及腹泻的发生率显著升高。因此，在PI3K抑制剂的应用过程中，应警惕相关不良反应的发生，必要时可予预防性抗感染治疗。约20％的FL患者合并EZH2突变，EZH2抑制剂是目前FL治疗中的重要靶向药物。研究显示，POD24人群接受EZH2抑制剂的ORR分别为63％（突变型）和25％（野生型），与总人群疗效相似[15]。提示EZH2抑制剂也可以考虑应用于POD24患者中。

目前研究结果显示细胞免疫疗法，包括嵌合抗原受体T（CAR-T）细胞治疗及双特异性抗体在POD24患者中取得较高ORR和CR率。ZUMA-5研究患者中有55％为POD24患者，Axi-cel在POD24患者的CR率达72％[16]。ELARA研究纳入患者中有62.9％为POD24患者，tiso-cel在POD24患者的CR率为59％[17]。双特异性抗体（CD20×CD3）在POD24患者也显示比较高的CR率。Mosunetuzumab 的Ⅰ期临床研究纳入62例R/R FL患者，其中48％为POD24患者。在POD24患者中，53％取得持续CR，而总体则为50％的持续CR率。提示双特异性抗体有望在POD24患者中获益。多项研究还显示双特异性抗体联合治疗，疗效会进一步提高。本中心回顾性研究显示，双特异性抗体单药在R/R FL的ORR为75％～85％，而联合治疗（如联合R2方案）则ORR可提升至90％以上[18]。总体来讲，细胞免疫疗法对POD24患者疗效要好于小分子靶向药物，本中心这例患者应用CD20×CD3双特异性抗体的联合治疗（Mos-R）取得比较满意疗效。

二、多线复发FL患者的治疗

1. 典型病例：男，60岁。因“发现颈部右侧肿物2个月”于2018年7月就诊，行颈部右侧肿物活检，术后病理（右颈部）诊断为FL，3A级。免疫组化：CD20（+），BCL2（+），CD10（−），MUM-1（−），CD23（滤泡网存在伴扩大），BCL6（+），MYC（−），Ki-67（50％+）。骨髓活检未见淋巴瘤累及。PET-CT：FL累及颈部双侧、纵隔、双肺门、双侧腋窝、腹膜后、盆腔淋巴结，累及脾脏；右侧髂骨等局部骨髓代谢稍升高，肿瘤累及待排。诊断：FL（3A级，Ⅲ期A，FLIPI 2分，FLIPI-2 1分），根据中国滤泡淋巴瘤诊断及治疗共识[19]，患者无治疗指征，基于患者要求及延长需要再次治疗时间的目的，予以BR单药×8次免疫治疗，治疗后颈部肿物消失，获得CR。2020年11月无明显诱因自觉颈部、腹股沟淋巴结肿大，行右腹股沟淋巴结活检。术后病理（右侧腹股沟淋巴结）诊断为FL，3A级，完善疾病分期诊断FL（3A级，Ⅳ期A，FLIPI 2分，FLIPI-2 1分），考虑病情进展，且超过3个淋巴结区的淋巴结长径>3 cm，达到治疗指征，R-CEOP×6方案化疗达到CR。并予BR方案维持治疗。2022年10月再次出现颈部右侧淋巴结肿大，颈部淋巴结活检示（颈部右侧淋巴结）FL，3A级。诊断FL（3A级，Ⅳ期A，FLIPI 2分，FLIPI-2 1分），予加用PI3K抑制剂（林普利塞）治疗，半年后达到CR并持续至今。

2. 多线复发FL患者的治疗现状：随着复发线数的增加，FL患者的生存显著下降。2019年，Rivas-Delgado等[20]回顾2001年至2014年接受免疫化疗的348例初诊FL患者的远期预后。研究显示，接受治疗线数越多的患者，中位OS期显著缩短。全部人群中有111例患者接受二线治疗，以及41例患者接受三线治疗。一线患者的中位OS期未达到，二线和三线患者的中位OS期分别为7.6和4.8年。

为探讨多线复发FL的临床特征和治疗现状，2022年Casulo等[21]进行多中心队列研究，评估既往接受≥3线全身治疗的441例FL患者的治疗模式和结局，其中13％患者既往接受auto-HSCT。研究显示，这组人群仍有较高的生存率。中位随访时间为71个月，3线以上复发患者的5年OS率为75％，中位PFS期为17个月。这组患者的治疗模式差异巨大，不同治疗方案的有效率也不同，但均有高反应率、低缓解持续时间的特点。仅从数据分析，免疫化疗（包括细胞治疗）的ORR和2年PFS率均是最高的。

3. 多线复发FL的治疗进展：针对多线复发FL的治疗，美国国立综合癌症网络指南（NCCN）主要推荐为靶向药物（PI3K抑制剂及EZH2抑制剂）及细胞治疗（双特异性抗体及CAR-T细胞治疗）。

在目前国内可及的2种PI3Kδ抑制剂均是获批用于≥3线治疗的R/R FL患者，ORR为80％～83％，CR率为26％～30％，PFS期为9.5～13.4个月。EZH2抑制剂单药用于≥3线R/R FL患者的研究显示，EZH2突变组患者ORR为69％，中位PFS期为13.8个月，而EZH2未突变组的ORR为35％，中位PFS期为11.1个月[15]，也被NCCN指南推荐用于R/R FL的三线治疗。

目前正在研究中的CD20×CD3双特异性抗体包括mosunetuzumab、glofitamab、epcoritamab及odronextamab等，均在多线R/R FL患者中取得良好疗效。其中，mosunetuzumab治疗R/R FL的Ⅱ期临床试验中，CR率为60％。不良反应方面细胞因子释放综合征（Cytokine release syndrome, CRS）发生率为44％，但主要为1级（26％）或2级（17％），通常仅发生在第1周期[22]。另外epcoritimab治疗R/R FL的Ⅰ/Ⅱ期临床试验中，FL的ORR为90％，CR率为50％。59％的患者发生CRS，但均为1～2级[23]。目前，mosunetuzumab已被美国NCCN指南推荐用于R/R FL的三线治疗。

CAR-T细胞治疗同样在R/R FL患者中取得良好疗效。其中，ZUMA-5研究显示Axi-cel在124例R/R FL患者中，ORR为94％，CR率为77％，中位DOR未达到；3～4级CRS发生率为7％，3～4级免疫效应细胞相关神经毒性综合征（Immune effector cell-associated neurotoxicity syndrome, ICANS）发生率为19％[16]。ELARA研究入组R/R FL患者97例，tiso-cel治疗的ORR 86.2％，CR率69.1％；无3～4级CRS发生，3～4级ICANS的发生率3.3％[17]。在中国开展的RELIANCE研究提示，28例R/R FL患者接受瑞基奥伦赛的治疗，ORR为100％，CR率为92.6％，同时≥3级CRS和ICANS的发生率分别为0及3.6％[24]。目前，CAR-T细胞治疗已被美国NCCN指南推荐用于R/R FL的三线治疗。而中国2023年滤泡淋巴瘤诊断与治疗专家共识中，瑞基奥伦赛也被推荐用于R/R FL的三线治疗[19]。

目前国内可及的用于多线复发FL的药物主要为PI3K抑制剂、EZH2抑制剂、CAR-T细胞以及临床研究。需综合考虑患者体质、疾病情况、既往治疗方案及经济等因素进行选择，以较小的代价获得较长的生存时间和较好的生活质量。

三、转化FL的治疗

1. 典型病例：男，46岁，主因“发现腹部肿物1个月余”于2020年2月17日入院。2020年1月体检发现腹部肿物，约10.7 cm×8.6 cm×6.8 cm。2020年2月3日PET-CT示：左上腹肿瘤性病变，腹膜后、肠系膜多发性淋巴结累及（SUVmax 11.2）。完善腹膜后肿物穿刺，（腹膜后肿物）符合FL，2级。免疫组化结果：CD20（+），CD10（+），Ki-67（30％+），BCL6（+），MUM-1（−），BCL2（+），C-MYC（5％+），CD21（滤泡网+）。骨髓活检未见淋巴瘤累及。诊断：FL（2级，Ⅰ期A，FLIPI 0分 FLIPI-2 1分），因腹腔包块>7 cm，达到治疗指征，建议启动治疗，患者要求考虑。2020年6月复查彩超左上腹见低回声团块，大小约14.0 cm×8.2 cm×10.0 cm，于2020年6月至2021年1月共予苯达莫司汀联合BR方案化疗6个疗程。中期评估疗效部分缓解。

2021年2月出现尿频尿急，进行性加重，抗感染疗效不佳，进行性腰酸伴血尿，2021年2月23日PET-CT：①原左上腹软组织肿块较前明显缩小、代谢减低；原腹膜后、肠系膜多发高代谢肿大淋巴结较前缩小、代谢减低，局部残留少许高代谢结节灶。②前列腺区周围软组织病灶，SUVmax 13.3。完善前列腺活检考虑DLBCL，GCB来源，CD20（弥漫+），Ki-67（80％+），BCL2（+），CD10（+），BCL6（+），MUM-1（−），C-MYC（40％弱+），FISH检测示MYC及BCL2阳性，同时合并TP53突变。诊断：高级别B细胞淋巴瘤，检查期间患者进行性无尿，血肌酐最高达269 µmol/L，考虑肿瘤相关性肾后性梗阻。2021年3月26日开始予R-DA-EPOCH方案（BR、依托泊苷、表柔比星、长春新碱、环磷酰胺、泼尼松）化疗，尿少症状改善，肌酐降至正常，但出现Ⅳ度骨髓抑制，4月21日再次应用R-DA-EPOCH方案，但疗效不佳，再次出现少尿，考虑肿瘤进展，化疗不敏感，备行CAR-T细胞治疗。5月12日换用R-DHAP（BR、阿糖胞苷、顺铂、地塞米松）治疗症状缓解后，采集干细胞及淋巴细胞。7月完成CAR-T细胞治疗联合auto-HSCT，CRS 1级。至今维持完全缓解。

2. 转化FL患者的治疗现状：每年有2％～3％ FL患者将转化为更高级别肿瘤，最常见类型为DLBCL[25]。回顾性研究显示，BR方案治疗出现FL患者中，约13％患者出现POD24，这一数据低于既往R-CHOP方案报道，但BR后出现POD24的人群合并转化比例明显升高，为76％。10％～25％的tFL患者可转化为“双打击”（合并BCL2和MYC重排）淋巴瘤，导致预后不良。根据中国多中心回顾性研究，tFL患者的生存率显著下降，5年OS率约为70％[1]。

针对tFL的治疗，若患者为初诊FL合并DLBCL成分或未经治疗出现转化，应按照初诊DLBCL治疗，大部分患者临床应答率良好。如患者合并“双打击”，应注意调整为R-DA-EPOCH为基础的方案并注意预防中枢神经系统复发。而对于接受免疫化疗后转化的FL患者，推荐方案参照难治复发DLBCL，主要循证医学证据集中于二线挽救化疗序贯auto-HSCT及CAR-T细胞治疗[26]。回顾性分析均显示，30％～50％的tFL患者可通过二线挽救化疗序贯auto-HSCT达到治愈。其中，2023年获批上市的维泊妥珠单抗联合BR的pola-BR方案在难治复发DLBCL中显示出良好疗效。一项来自希腊的多中心回顾性研究发现，<65岁因化疗不敏感而不适合auto-HSCT的复发难治DLBCL患者，经pola-BR方案治疗后，约40％患者可转变为潜在适合auto-HSCT[27]。而桥接auto-HSCT则可使tFL患者的生存显著优于未接受auto-HSCT的患者[26]。异基因造血干细胞移植（allo-HSCT）目前在tFL中的地位仍有争议。回顾性研究显示，在33例接受allo-HSCT的tFL患者中，中位随访时间为7.1年，患者5年OS率为33％。化疗敏感、减低剂量预处理及慢性GVHD发生率低是患者OS良好的预后因素[28]。目前allo-HSCT主要用于auto-HSCT后复发或CAR-T细胞治疗后复发的年龄患者。此外，CAR-T细胞成为治疗tFL的“利器”。研究显示，CAR-T细胞治疗在tFL患者可取得高缓解率。其中ZUMA-1研究入组24例tFL患者，ORR达到80％，DOR为8.2个月[29]。而JCAR017研究入组14例tFL患者，ORR达到75％[30]。上述数据提示，在化疗不敏感的tFL患者中，CAR-T细胞治疗选择值得考虑。CAR-T细胞联合auto-HSCT治疗侵袭性淋巴瘤的研究入组了4例tFL患者，研究显示CAR-T联合auto-HSCT可使患者的生存获益进一步优于单独CAR-T细胞治疗[31]。

综上，FL是最常见的惰性淋巴瘤，总体的生存期较长，但早期进展、转化或多线复发的患者预后不良。目前有多种新型疗法，包括小分子靶向药物、双特异性抗体及CAR-T细胞等显著改善这部分FL患者的预后，应根据患者疾病特征、体能状态及既往治疗方案，个体化选择最合适的治疗方案，将有助于临床的最大获益。
